# The Birth of the Eye Vesicle: When Fate Decision Equals Morphogenesis

**DOI:** 10.3389/fnins.2018.00087

**Published:** 2018-02-21

**Authors:** Florence A. Giger, Corinne Houart

**Affiliations:** Department of Developmental Neurobiology, Centre for Developmental Neurobiology and MRC Centre for Developmental Disorders, Institute of Psychiatry, Psychology & Neuroscience (IoPPN), King's College London, London, United Kingdom

**Keywords:** forebrain morphogenesis, neurulation, eye vesicle, compartment boundary, cell movement, cyclopia

## Abstract

As the embryonic ectoderm is induced to form the neural plate, cells inside this epithelium acquire restricted identities that will dictate their behavior and progressive differentiation. The first behavior adopted by most neural plate cells is called neurulation, a morphogenetic movement shaping the neuroepithelium into a tube. One cell population is not adopting this movement: the eye field. Giving eye identity to a defined population inside the neural plate is therefore a key neural fate decision. While all other neural population undergo neurulation similarly, converging toward the midline, the eye field moves outwards, away from the rest of the forming neural tube, to form vesicles. Thus, while delay in acquisition of most other fates would not have significant morphogenetic consequences, defect in the establishment of the eye field would dramatically impact the formation of the eye. Yet, very little is understood of the molecular and cellular mechanisms driving them. Here, we summarize what is known across vertebrate species and propose a model highlighting what is required to form the essential vesicles that initiate the vertebrate eyes.

The eye mainly comprises the retina pigment epithelium (RPE), retina, lens and external accessory tissues (iris and cornea). The earliest morphogenetic event strictly required for eye formation is the development of the eye vesicle (progenitors of optic stalk, retina, and RPE), evaginating from the neural tube. This outpocketing is required for induction of ectodermal-derived outer tissues (lens, iris, and cornea). This essay focuses on the early determination and morphogenetic events leading to the formation of two bilateral eye vesicles.

## Emergence of eye field identity inside the anterior neural plate

The initial neural epithelium, called the neural plate, is specified during gastrulation from the dorsal ectoderm. A key step in neural plate induction is the inhibition of Bone Morphogenetic Protein (BMP) signaling, widely active in blastula and progressively repressed, from medio-posterior to latero-anterior, throughout gastrula stages by BMP antagonists emitted by the Spemann Organizer (node in amniotes, dorsal lip of the blastopore in *Xenopus* and shield in fish). Signaling by Fibroblast Growth Factors (FGFs), Insulin-like Growth Factors (IGFs), Wnts and Wnt inhibitors are also implicated early in this process (Wilson et al., 2001; Wessely and De Robertis, 2002; Pera et al., 2003; De Robertis and Kuroda, 2004; Fuentealba et al., 2007; Anderson and Stern, 2016). Some studies indicate that neural induction begins before onset of gastrulation, when ectodermal cells are primed to become responsive to the neural-inducing signals mentioned above (Linker and Stern, 2004; Albazerchi and Stern, 2007; Pinho et al., 2011).

**Figure 1:**
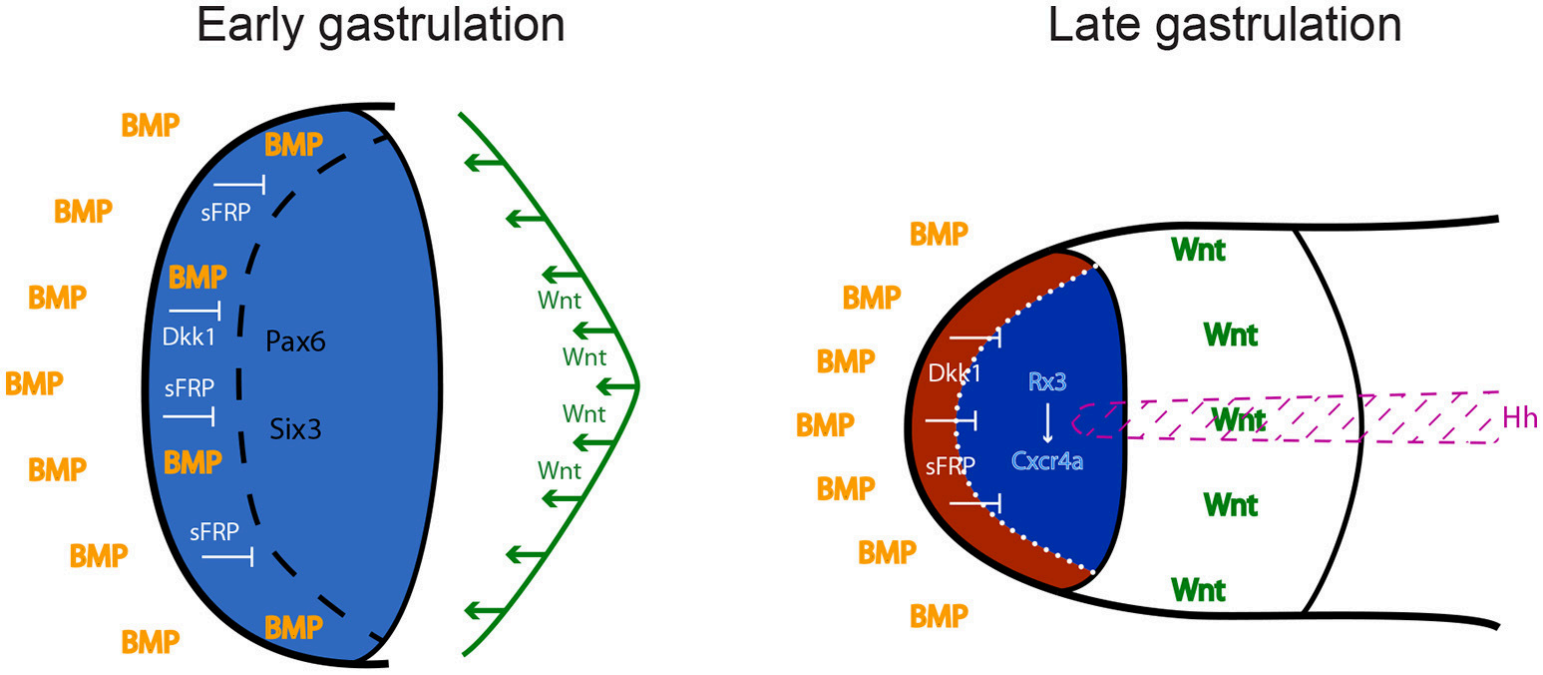
Anterior neural plate patterning during gastrulation. In order to acquire eye identity, BMP signals have to be restricted anteriorly, and posteriorising Wnt ligands have to be antagonized by sFRPs and Dkk1. Blue: forebrain territory (telencephalon and eye field). Red: telencephalon; dark blue: eye field. Hh: Hedgehog; BMP: Bone Morphogenetic Protein; sFRPs: secreted Frizzled Receptor Proteins; Dkk1: Dickkopf 1.

The neural plate is patterned in distinct subdomains from anterior to posterior: the forebrain (or prosencephalon), midbrain, hindbrain, and spinal cord. The forebrain comprises telencephalon, eye and diencephalon. Classical studies in amphibian embryos suggested that neural induction *per se* generates tissue of anterior neural character, and that posterior neural identity is subsequently imposed by a factor called the “transforming signal” (Nieuwkoop et al., 1952; Stern, 2001). Later studies identified this postulated signal as a combination of FGFs, retinoic acid and Wnts (Maden, 2002; Niehrs, 2004; Mason, 2007; Bielen and Houart, 2014). According to this view, the forebrain, including the precursors of the eye field, is induced in an area of the neural plate that is devoid of these posteriorising instructive factors. Wnts appear to play a particularly important role in antagonizing anterior neural fates. Several inhibitors of the Wnt pathway are released by tissues that are in close proximity to the future forebrain region: Cerberus, Dickkopf1 (Dkk1) and Frzb1 are secreted by the anterior mesendoderm that underlies the prosencephalon, and the anterior neural border of the frog and zebrafish embryo produces several Soluble Frizzled-Related Proteins including Crescent, SFRP1, SFRP5, and Tlc (Niehrs et al., 2001; Houart et al., 2002; Tendeng and Houart, 2006). Loss-of-function experiments in frog, mouse and zebrafish embryos demonstrated that Dkk1 and/or SFRPs are required for forebrain formation (Glinka et al., 1998; Mukhopadhyay et al., 2001; Houart et al., 2002). Wnt/β-catenin plays a central role in forebrain patterning, promoting diencephalic at the expense of telencephalic/eye field fates (Houart et al., 2002; Braun et al., 2003; Wilson and Houart, 2004). This indicates that differences in timing and/or specific doses of the Wnt signal are crucial for the establishment of different forebrain subdivisions, although not implicated in the fate distinction between the anterior-most features telencephalon and eye field, territories both devoid of Wnt activity (Figure 1).

Contradicting the dogma defining anterior neural fate as “default,” developing from absence of signaling activities, BMP signaling is actually required during early-mid gastrulation to subdivide the anterior prosencephalic field into telencephalon and eye field (Figure 1). Zebrafish studies demonstrated that this cell fate choice is driven by spatiotemporally-controlled P-Smad1/3/5 activity, which represses the induction of eye specification factors in the prospective telencephalic domain, thereby preventing it from adopting retinal identity (Bielen and Houart, 2012).

Secreted signaling factors organize the neural plate along the anteroposterior axis. This pattern is translated into combinatorial codes of transcription factor expression. These codes translate specific doses and/or combinations of signaling activities into distinctive cell fates that are subsequently reinforced and converted into specific cellular behaviors. Anterior transcriptional determinants that are antagonized by Wnts include the homeobox genes OTX2 (expressed in forebrain and midbrain), PAX6 (forebrain only), HESX1, and SIX3 (anterior forebrain). Genetic disruption of each of these factors results in varying degrees of forebrain defects (Acampora et al., 1995; Matsuo et al., 1995; Dattani et al., 1998; Lagutin et al., 2003; Andoniadou et al., 2007; Georgala et al., 2011).

OTX2 is expressed early in the prospective forebrain and is required for the expression of PAX6, SIX3, and RX/RAX, three major regulators of eye development. OTX2 is subsequently down-regulated by these factors during eye specification (Andreazzoli et al., 1999).

PAX6 is expressed in the presumptive anterior brain from flies to mammals (Walther and Gruss, 1991). Its misexpression in Drosophila and *Xenopus* leads to ectopic eye structures (Halder et al., 1995; Kenyon et al., 2001), and loss-of-function leads to reduction of the eye, including the ectoderm-derived lens (Quiring et al., 1994; Halder et al., 1995; Macdonald and Wilson, 1996). Recent studies using CRISPR/Cas9 in mouse embryo to create mosaic expression of PAX6 mutation have enabled to investigate the dosage requirement of PAX6 for eye development and show that the development of the lens from the surface ectoderm requires a higher dose of PAX6 than retinal maturation inside the optic vesicle (Yasue et al., 2017).

SIX3 is expressed in the anterior brain from Drosophila to mammals (Oliver et al., 1995; Loosli et al., 1998; Seo et al., 1998). Its misexpression results in ectopic retina and lens formation in mouse and medaka through ectopic induction of RX/RAX (Loosli et al., 1999). Loss-of-function analyses in medaka and mouse have demonstrated that SIX3 plays a key role in establishment of forebrain fate in the neural plate, including retinal identity (Carl et al., 2002; Lagutin et al., 2003).

RX/RAX is a well-conserved essential homeobox protein initially expressed in the eye field, then in the budding bilateral eye vesicles. RX/RAX misexpression induces ectopic eye formation in *Xenopus*. Fish (zebrafish and medaka) and mouse homozygous loss-of-function mutants do not exhibit any eye structure, demonstrating that this protein is critical for eye formation in vertebrates (Mathers et al., 1997; Loosli et al., 2003; Stigloher et al., 2006). The first interpretation of RX loss-of-function studies in Medaka was that eye field cells lacking RX were keeping their identity but were trapped inside the forebrain rod (Loosli et al., 2003). Stigloher et al. subsequently found in zebrafish that RX3-deficient eye field cells were in fact expressing a telencephalic program concomitantly to some eye specification markers. They further demonstrated that RX3 imposes retinal identity to anterior forebrain cells by actively repressing telencephalic programmes prior to neurulation (Stigloher et al., 2006). Furthermore, RX3 needs to be repressed by P-Smad1/5/8 activity in the anterior part of the *six3*-positive field to enable the establishment of a telencephalon territory (Bielen and Houart, 2012). Together, these findings highlight the key role of RX3 in eye field/telencephalon differentiation process.

## Separation of a single eye field in two bilateral eye primordial

The immediate consequence of the expression of the eye field transcriptional code is the trigger of distinct cell behavior leading to the formation of eye vesicles.

### Separation of the eye field by medial repression of eye identity

The prechordal axial mesoderm underlying the anterior neural plate secretes Nodal ligands that directly induce *sonic hedgehog* (shh) expression in the axial neural epithelium (Müller et al., 2000). This axial population is forming the hypothalamus anteriorly, directly above the prechordal plate. SHH mutants display cyclopia, demonstrating that Hedgehog signaling from the hypothalamus is necessary for eye field separation (Ekker et al., 1995; Macdonald et al., 1995; Chiang et al., 1996). Hedgehog is required to impose hypothalamic fate to the initial medial eye field. Nodal signaling being required for the formation of a prechordal plate, Nodal-deficient zebrafish mutants *squint, cyclops* and one *eyed pinhead* fail to form hypothalamic progenitors. Being deprived of SHH source, Nodal-deficient mutants don't separate the eye field, thus exhibiting cyclopia (Hatta et al., 1991; Feldman et al., 1998; Sampath et al., 1998; Gritsman et al., 1999).

Cyclopia can also occur after eye evagination. At that stage, SHH induces the optic stalk marker *pax2* and represses the retinal marker *pax6*. The loss of *pax2* expression triggers the expansion of *pax6* expression medially, inducing retinal fate at the expense of optic stalk fate, leading to fusion of the bilateral eye vesicles (Ekker et al., 1995; Macdonald et al., 1995). Post neurulation, SIX3 is required to maintain *shh* expression, which in turn maintains *six3* expression in the diencephalon, in a positive regulatory loop. Loss of *six3* expression in the nascent neural tube results in loss of *hedgehog* expression, and therefore in the failure to separate the eye field (Geng et al., 2008; Jeong et al., 2008).

### Cellular movements during gastrulation

In *Xenopus*, cells populating the retina come from nine animal blastomeres in the 32-cell embryo (Huang and Moody, 1993). Low levels of BMP signaling are required for animal blastomeres to contribute to the retina (Moore and Moody, 1999). Animal blastomere neural progenitors competent to acquire eye identity disperse and populate the retinal territory. In the blastula, the dispersion of clones populating the eye field territory is dependent on FGFR2 and EphrinB1 reverse signaling pathway (Moore et al., 2004). This is mediated by the Wnt/PCP pathway, the intracellular part of EphrinB1 associating with the DEP domain of Disheveled (Lee et al., 2006).

In zebrafish, progenitors at the midline move anteriorly inside the neural plate during convergence/extension, thereby adopting their appropriate AP positions along the elongating neuraxis (Chuang and Raymond, 2002). These gastrulation movements alter the initially uniform shape of the eye field, developing a posterior, median indentation (Varga et al., 1999). This indentation exists in all vertebrate anterior neural plates studied and is necessary to divide the eye field into two eye primordia (Varga et al., 1999; Moore et al., 2004).

### Physical bisection by the prospective diencephalon

In addition to the inductive role of the prechordal plate leading to a repression of eye identity medially, the anterior migration of the prechordal plate also promotes the anterior-ward movement of axial diencephalic progenitors (future hypothalamus).

Micro-ablation experiments in zebrafish have demonstrated that diencephalic progenitors migrate through the eye field, thus physically splitting it in two bilateral eye fields (Varga et al., 1999; Hirose et al., 2004; England et al., 2006). Although one cannot exclude the possibility of “sheering” movement inside the medial neuro-epithelium of non-teleosts, the strong epithelialisation of their neural plate makes this event less likely. Micro-dissection experiments in chick and *Xenopus* have demonstrated that the eye field separation is dependent on the anterior-ward migration of the prechordal plate (Li et al., 1997; Pera and Kessel, 1997). However, this movement is necessary to move Nodal and Hh-secreting cells forward underneath the medial neural plate, themselves involved in repressing eye and/or promoting medial diencephalic (hypothalamus) identity.

Separation of the eye field thus likely results from the combination of physical bisectioning axial movements and genetic repression of retinal fate medially, ensuring robustness of this fundamental process.

## Establishment of boundaries enabling differential movements during anterior neurulation

**Figure 2:**
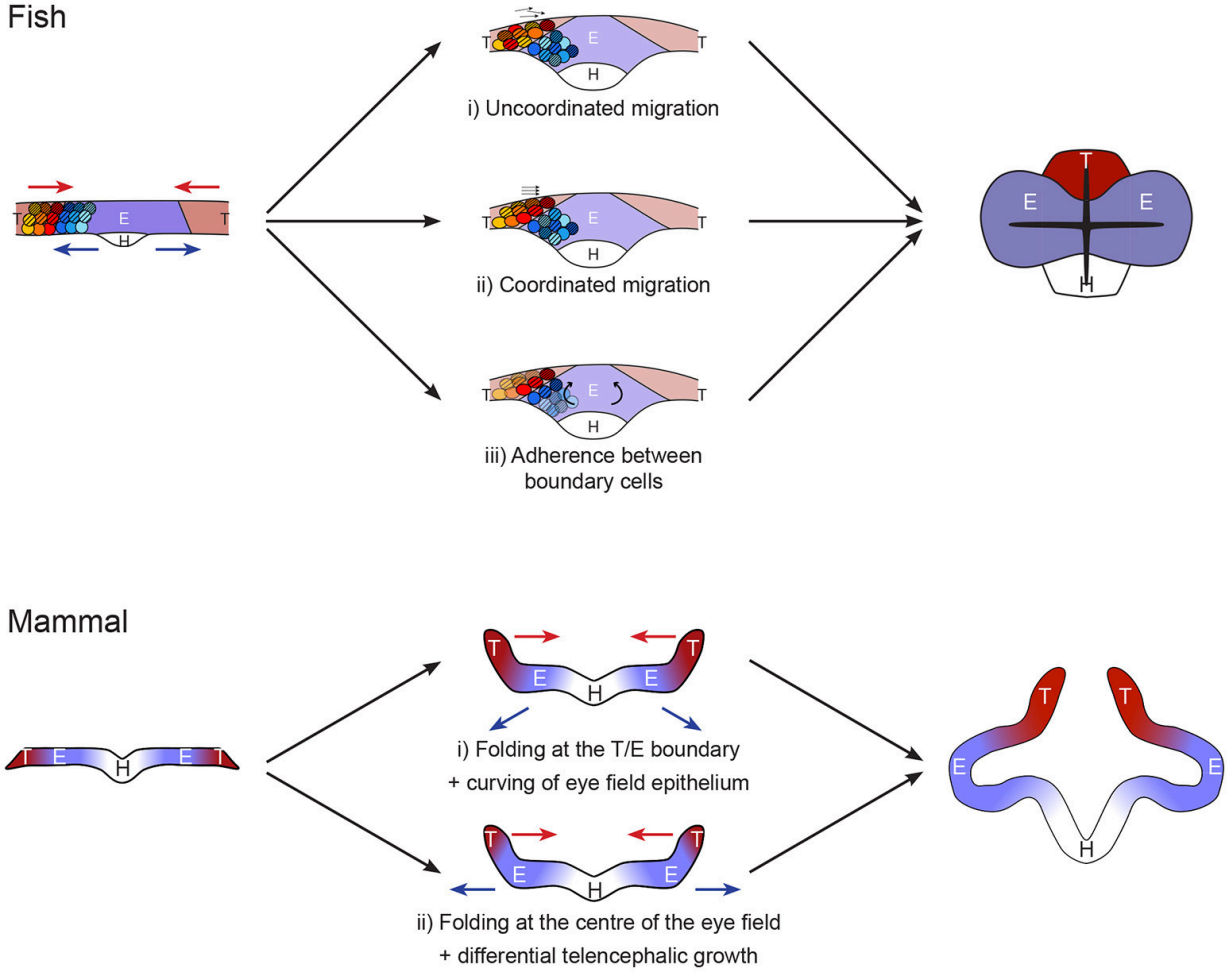
Models of forebrain neurulation from fish to mammals. Fish: telencephalic cells migrate above the eye field without mixing. This could be achieved by either (i) uncoordinated or (ii) coordinated telencephalon migration, independently of eye field cell behavior. (iii) Alternatively, the movement may be driven by folding, through rotation of eye field cells. This mode requires strong adhesion between telencephalon and eye at the boundary as well as inside the telencephalic population. Mammal: Studies are very sparse. Absence of marker analyses precludes strong statement on the exact position of the boundaries between hypothalamus, eye field and telencephalon. The initial folding of the neural plate may occur (i) at the boundary between eye field and telencephalon or (ii) in the middle of the eye field. Scenario (i) resembles the fish situation, in which the telencephalon converges toward the midline while the eye field resists these neurulation movements. This step is then followed by deformation of the eye field by apical constriction of its center. Alternatively, in ii) the folding of the neural plate at the middle of the eye field comes first, followed by differential growth of a small telencephalic initial primordium.

Once the eye field, hypothalamus and telencephalon territories have been specified, cells from these domains undergo very different morphogenetic movements: the hypothalamic population adopts a v-shape structure, the telencephalic cells move toward the midline while eye progenitors maintain their position before moving outwards for the formation of the eye vesicles (Figure 2; Keller et al., 1992; Zolessi and Arruti, 2001; England et al., 2006; Rembold et al., 2006). Despite these very distinct dynamic events, no cell mixing is observed at the borders between eye field and telencephalon dorsally or hypothalamus ventrally. Fate specification drives these distinct complex morphogenetic movements and elaborates the formation of strict tissue boundaries. The maintenance of distinct territories and coordinated tissue folding during development has been shown to rely on a combination of strong adhesion and contact inhibition (Dahmann et al., 2011; Fagotto, 2015). All these are essential to the initial evagination of eye primordia during anterior neurulation.

### Strong eye field adhesion

The mechanisms driving the distinct behavior of the eye field from the rest of the neural epithelium during neurulation are mostly uncharted waters, despite the crucial importance of this process for normal forebrain and visual system development. One main reason for this gap in knowledge is the difficulties to study cellular behaviors at these early stages of development in most model organisms. For this reason, most of the progress in understanding has been made in zebrafish and frog, amenable to imaging of early developmental stages.

In the eye field of zebrafish, the Wnt/β-catenin signaling pathway is antagonized in part by Wnt non-canonical pathway (Cavodeassi et al., 2005). In addition to its patterning role, Wnt non-canonical signaling induces cell adhesion, which maintains coherence of the eye field. In zebrafish, lack of Wnt11 (*slb* mutant) or its eye field specific receptor Fz5 leads to defective morphogenesis of the forebrain and results in delayed eye field evagination and smaller eyes (Cavodeassi et al., 2005; England et al., 2006). In *Xenopus*, the Wnt/PCP pathway has been shown to cooperate with the Eph/Ephrin signaling pathway during eye field morphogenesis (Lee et al., 2006). Fzd5 is expressed in the eye field at E8.5 in the mouse, supporting a similar role in mammals (Kemp et al., 2007).

In zebrafish, the chemokine receptor Cxcr4a is required to maintain the boundaries between the eye field and telencephalon/hypothalamus. Cxcr4a is expressed specifically in the eye field, downstream of Rx3, and prevents cell mixing at the junctions between eye field and the rest of the neural plate during neurulation (Bielen and Houart, 2012). Cxcr4a has been shown to promote cell adhesion in different contexts (Hartmann et al., 2005; Engl et al., 2006; Nair and Schilling, 2008), suggesting that its segregating role in the forebrain may be mediated by the promotion of strong adhesion within the eye field during telencephalon migration.

### Boundary between the eye field and adjacent territories

The Eph/Ephrin signaling has been involved in the formation of boundaries in a number of embryonic structures such as the hindbrain (Gilardi-Hebenstreit et al., 1992; Xu et al., 1995) and somites (Durbin et al., 1998; Watanabe et al., 2009). Eph receptors constitute a large family of receptor tyrosine kinases binding Ephrin ligands. Both receptors and ligands being attached to the cell membrane, Eph/Ephrin signaling requires cell contact. In the majority of cases, Eph signaling causes cell repulsion away from the Ephrin-expressing cell, although adhesive responses have also been described (Kullander and Klein, 2002; Pasquale, 2008).

Complementary patterns of expression of Eph receptors and Ephrin ligands have been described in the forebrain in fish and *Xenopus* (Xu et al., 1996; Jones et al., 1997). In fish, EphA4a receptor is expressed in the telencephalon while Ephrinb2a ligand is expressed in the eye field (Xu et al., 1996; Cooke et al., 1997; Cavodeassi et al., 2013). This complementarity pattern is dependent on regional specification, being lost in *rx3/chk* mutants (Cavodeassi et al., 2013). Disruption of Eph/Ephrin signaling induces a delay in optic vesicle expansion and cell intermixing from the eye territory with the adjacent telencephalon and diencephalon territories, demonstrating that Eph/Ephrin signaling is important to maintain cell segregation between adjacent domains during forebrain morphogenesis (Xu et al., 1996; Cavodeassi et al., 2013).

The differential cellular behavior of the eye field inside the mature neural plate has not been explored in avian and mammalian models, restricting greatly our current understanding of this process. Further studies are required to determine the interplay between these different actors from fish to mammals. In addition, the potential deposition of extra-cellular matrix that would act as a physical barrier at the interface between the eye field and adjacent territories remains to be investigated.

What is known of the process has been mostly unveiled in a teleost, displaying a specialized neurulation. However, the conservation in expression of players such as Fzd5 (Kemp et al., 2007) suggests that some common mechanisms exist. It is therefore conceivable that a common molecular control of cell-cell interaction represses convergent neurulation movement in the eye field in all vertebrates and defines strong boundaries between this tissue and the rest of the anterior neural plate. In all vertebrates, the eye field population fails to adopt the neurulation movements initiated at the midline, adopted by the majority of the neural epithelium. Instead, the cell population keeps its mediolateral width, while the rest of the plate neurulates around it. The telencephalon/eye/hypothalamus boundary cells allow telencephalon neurulation by adopting a very specialized morphology across vertebrates, although the nature of the telencephalic movement varies due to distinct constrains in fish and other vertebrate neural plates (mesenchymal-like vs. epithelial respectively). Differential proliferation is predicted to play a bigger role in the process in non-teleosts (Figure 2).

## Eye vesicle evagination

Formation of eye vesicles has first been described by the precise observation of cell shape with electron microscopy in mice: specialized optic vesicle cells inside the closing neural plate first become columnar, then wedge-shaped following constriction of the cell apices to form a C-shaped vesicle. Cells elongate 2 times their initial height before the neural tube fully closes, then shorten as the vesicle is completed. Cell apices decrease in width. The formation of eye vesicles is accompanied by uneven deposition of basal lamina that thus appears patchy (Svoboda and O'Shea, 1987).

During eye vesicle evagination, β-catenin has been shown to accumulate at the luminal surface in the central optic primordium in rat. Interference with GSK3-β induces lack of membrane β-catenin accumulation, as well as deficient optic vesicle formation, lack of cell proliferation and continuity in basal membrane. This suggests that β-catenin accumulation could induce disruption of the basal membrane and increase of cell proliferation that in turn would lead to morphogenetic changes driving the evagination of the optic primordia (Matsuda and Keino, 2001).

Live imaging in fish embryo described complex cell movements during eye vesicle formation: prior to the onset of optic vesicle evagination, posterior eye cells are drawn deep and anteriorwards. Anterior and lateral eye field fold toward the midline and thus position above medial and posterior eye cells. Basally positioned cells acquire apico-basal polarity in a Laminin-dependent manner and establish a pseudostratified neuroepithelial organization, while apical cells remain mesenchymal. Lateral retinal progenitors then migrate laterally into the evaginating optic vesicles. At later stages, apical cells undergo a mesenchymal-to-epithelial transformation during which they elongate and intercalate between cells of the epithelialised domain of the eye field, thereby contributing to the later steps of tissue evagination. Very little cell proliferation is involved in the fish during eye vesicle formation (England et al., 2006; Rembold et al., 2006; Ivanovitch et al., 2013), in contrast to amniotes epithelium that undergoes dramatic growth at that stage.

Mammalian optic cups can self-organize *in vitro* from three-dimensional embryonic stem cell culture, and Laminin is required for this process. This self-organization shows that vesicle morphogenesis can occur intrinsically from a spherical neuroepithelium, without any requirements for external signals. In addition to providing new ways to decipher eye formation, the creation of these organoids opens up opportunities for regenerative medicine and retina transplantation (Eiraku et al., 2011; Nakano et al., 2012).

## Concluding remarks

Genetic pathways conferring eye identity within the anterior neural plate have been well described. However, cellular mechanisms ensuring integrity of the eye field and then evagination into eye vesicles have not yet been elucidated. So far, cell movements in the forebrain have been mostly investigated in teleost fish, taking advantage of the amenability of this model for live imaging. Teleost fish forebrain organization is distinct from other species at the beginning of neurulation: rather than forming an epithelial sheet, anterior neural plate cells are still mesenchymal at this stage in fish. Interestingly, despite these differences, the critical event in all conditions is for the eye field to maintain its position, thereby resisting converging neurulation movements. This specific behavior is most likely to be instructed by genetic identity, determinants of which are largely conserved in vertebrates. This suggests that the key mechanisms enabling the eye field to resist neurulation movements may also be conserved. The current state of our knowledge leaves key questions unanswered and opens a series of hypotheses regarding the mechanisms at play across phyla, summarized in Figure 2. The emergence of super-resolution live imaging and highly effective genome editing tools now allow to test these hypotheses and will unravel the cellular and molecular mechanisms driving vesicle formation in the coming years.

## Author contributions

All authors listed have made a substantial, direct and intellectual contribution to the work, and approved it for publication.

### Conflict of interest statement

The authors declare that the research was conducted in the absence of any commercial or financial relationships that could be construed as a potential conflict of interest.
